# Electron field emission of water-based inkjet printed graphene films†

**DOI:** 10.1039/d5na00161g

**Published:** 2025-06-24

**Authors:** Alessandro Grillo, Towseef I. Ahmad, Jingjing Wang, Aniello Pelella, Enver Faella, Daniele Capista, Maurizio Passacantando, Antonio Di Bartolomeo, Cinzia Casiraghi

**Affiliations:** a Department of Chemistry, University of Manchester Manchester M13 9PL UK cinzia.casiraghi@manchester.ac.uk alessandro.grillo@manchester.ac.uk; b Physics Department “E. R. Caianiello”, University of Salerno Via Giovanni Paolo II n. 132, Fisciano 84084 Salerno Italy; c IHP Im Technologiepark 25 15236 Frankfurt (Oder) Germany; d Dipartimento di Scienze Fisiche e Chimiche, Università degli Studi dell'Aquila Via Vetoio, 67100 Coppito AQ Italy

## Abstract

Solution-processed graphene is extremely attractive for the realization of large area and patterned graphene films for field emitting devices. Previous studies have focussed only on the use of reduced graphene oxide; however, solution-processed graphene can also be produced by other approaches, giving rise to nanosheets with different surface chemistries and lateral and thickness distributions. Here, we report the field emission characterization of films made of water-based graphene ink, prepared by liquid phase exfoliation, and inkjet printed with an area of 2.5 mm^2^ on silicon (Si/SiO_2_) substrates. These films show excellent field emission properties, comparable to those measured on single flakes and carbon nanotubes with the same setup, and they show a remarkably high maximum current density (up to ∼723 A cm^−2^), making them very attractive for field emission devices.

## Abstract 2

Water-based inkjet-printed graphene films demonstrate exceptional field emission performance, enabling scalable, eco-friendly fabrication of advanced vacuum electronics.

## Graphical abstract

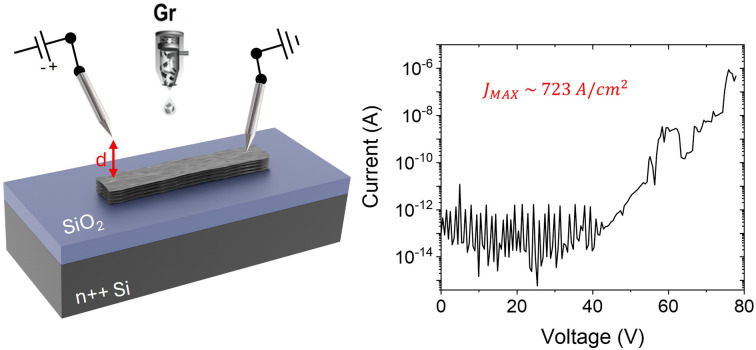

## Introduction

Since the initial demonstration of electron field emission from carbon nanotubes (CNTs),^1^ various low-dimensional carbon materials,^2^ such as nanorods,^3^ nanotips,^4^ nanofibers,^5^ nanowalls,^6^ and nanoflowers^7^ have undergone extensive investigation to explore their potential applications as electron sources. This interest is driven by their distinct structure and exceptional electronic and mechanical properties. In particular, CNTs exhibit promising electron-field-emission characteristics that surpass those of traditional field emitters, such as low turn-on voltage and high emission current.^8–10^ More recently, graphene has also attracted a lot of research attention as a field emitter due to its high aspect ratio, high electrical conductivity, and robust mechanical properties.^11–13^ In addition, previous research has highlighted that individual single- and few-layer graphene flakes are able to achieve turn-on fields of a few hundreds of V μm^−1^ from both the edge and the middle part of the flakes.^11,14,15^ However, single flakes are not suitable for large scale applications, hindering the possibility of using graphene in practical field emitting devices, such as flat-panel displays.^16^

Solution-processed graphene is very attractive for use in field emitting devices because the material can be deposited with mass scalable and cheap techniques into films with variable thickness and geometry on virtually any surface, without using high temperature processes, in contrast to chemical vapour deposition methods.^17,18^ So far, all the reported studies on the field emission of solution-processed graphene have focused on the use of reduced graphene oxide (r-GO). Previous research^16,19–21^ has demonstrated that field emission in graphene occurs at threshold fields comparable to those of CNT arrays, even for in-plane films, as the random orientation of the nanosheets in the film enables some of the flakes to be oriented at some angle with respect to the substrate surface and hence to contribute to the field emission.^16,19,20^ In particular, films of rGO have shown excellent emission uniformity and stability,^16,21,22^ attributed to the fact that the current is expected to be shared with atoms on line edges, in contrast to CNTs.^22^

As there is a family of graphene-based materials, characterized by different lateral sizes, thicknesses and functional groups,^23,24^ it is important to investigate the field emission properties of different types of graphene-based materials and the effect of the deposition technique, as this determines how the graphene nanosheets assemble together to form a film, including their orientation.^17,24^ This comparative analysis is crucial to understand the relationship between material properties and field emission performance. In the last few years, new methods for production of solution-processed graphene have been developed, such as liquid-phase exfoliation (LPE)^25^ and electrochemical exfoliation (ECE).^26–28^ LPE is a simple, low cost and scalable way to produce graphene by directly exposing the material to a solvent with a surface tension that matches the one of graphene. By adding suitable dispersing agents,^29^ it is possible to produce stable and highly concentrated water-based dispersions of high-quality graphene that do not require further processing (*e.g.* reduction) and that can be inkjet printed on several substrates, including plastic and paper.^30^ It is therefore interesting to compare the field emission properties of solution-processed graphene made by LPE with the ones obtained so far for rGO, having different lateral sizes, thicknesses and surface properties. Hence, this will enable getting some insights on how field emission is affected by defects and surface functionalization.

In this work, a water-based graphene ink, prepared by LPE, is inkjet printed onto a Si/SiO_2_ substrate to produce a film covering an area of 2.5 mm^2^. A tip-anode setup, realized inside a scanning electron microscope (SEM), is used to study the field emission properties of the film. We demonstrate the suitability of the printed graphene film as a field emitter, providing a remarkable maximum current density of ∼723 A cm^−2^ and stable emission for at least 3 hours, hence confirming the suitability of water-based and inkjet printable graphene inks for the realization of large-scale field emitting devices.

## Results and discussion

**Fig. 1:**
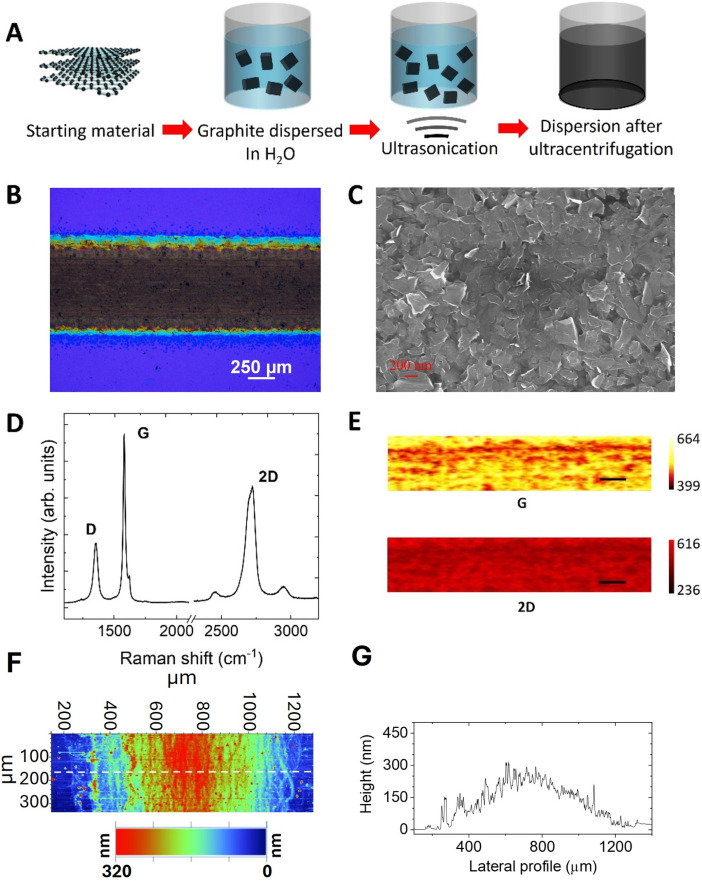
Graphene film morphology and properties. (A) Schematic of the liquid exfoliation process of graphene. (B) Optical image of the graphene film printed onto a Si/SiO_2_ substrate. (C) Top view SEM image of the printed graphene film. (D) Representative Raman spectrum of the printed graphene film. (E) G and 2D peak intensity maps obtained through Raman streamline mapping (the scale bar is 100 μm). (F) Profilometry map of the graphene film. The white dotted line represents the position where the single line profile has been measured. (G) Profile of the graphene film measured by single-scan profilometry.

Fig. 1A shows a schematic of the LPE of graphene used to obtain the water-based ink (see Methods for more details). The graphene ink is composed of nanosheets with an average lateral size and thickness of ∼170 nm and ∼6 nm, respectively.^31^ Note that the nanosheets are exfoliated with non-covalent functionalization in water, so the thickness of the nanosheet also includes contributions from the stabilizer molecules adsorbed on both sides of the flake.^31^ Previous characterization shows that the nanosheets have high crystallinity and they are C–O free.^32^ Graphene films were printed (see Methods for details) on doped n-Si (100) wafers covered with 300 nm thermally grown SiO_2_ (Fig. 1B). The graphene films have a length *L* of 5 mm and width *W* of 0.5 mm.

**Fig. 2:**
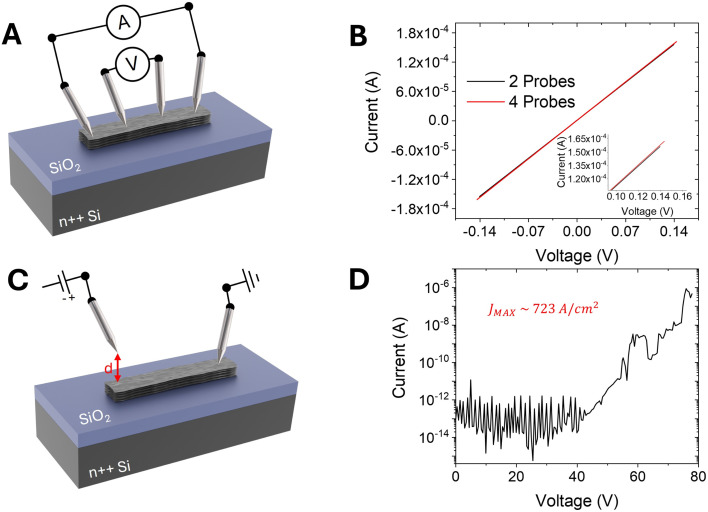
Graphene film electrical characterization. (A) Schematic of the electrical setup used to perform 4-probe characterization. (B) *I*–*V* curves recorded in both 2- and 4-probe setups. (C) Schematic of the electrical setup used to perform the field emission measurements. (D) *I*–*V* curve recorded with the field emission setup and an anode–cathode separation of 200 nm.

SEM imaging and Raman spectroscopy were employed to investigate the structure and the morphology of the printed films. Fig. 1C shows a representative SEM image of the printed graphene revealing a continuous and homogeneous film where most of the nanosheets have a planar orientation in agreement with the good electrical conductivity (see Fig. 2B). Fig. 1D shows a representative Raman spectrum of the graphene film, showing the typical features of films made using solution-processed graphene: a disorder-induced D peak at ∼1350 cm^−1^, a G peak at ∼1580 cm^−1^ and a 2D peak at ∼2680 cm^−1^.^25,30,33^ Streamline Raman mapping across a large area (1 × 0.2 mm^2^) further confirms the uniformity of the film: Fig. 1E shows that both the G peak and the 2D peak intensities are uniform across the entire area, confirming the lack of pinholes and areas not covered by the film. The thickness of the printed film, measured by using a stylus profilometer (see Methods), is ∼200 nm. Fig. 1F and G show an area and a line scan profile across the printed graphene film, respectively, showing that the film is characterized by a relatively high roughness – the estimated root mean square (RMS) roughness is 100 nm, indicating that the film has a very high aspect ratio, which is expected to be crucial for field emitters.^16^ This high aspect ratio resulted from the random restacking of nanosheets of different thicknesses when they assemble into a film.^34^ Fig. S1A and S1B† show 45° and 60° tilted SEM images, revealing that some graphene nanosheets clearly protrude from the film surface, creating favorable emission sites. A SEM image of the W-tip used for the field emission measurements is shown in Fig. S1C.† Because of the characteristic morphology of the printed graphene film, this material cannot be compared to planar graphene; in addition, as the film is composed of nanosheets of different thicknesses, the field-emission signal will be, in principle, given by the contribution of all the nanosheets.

To investigate the electrical properties, the devices with the graphene films were placed inside a SEM chamber and made to come into contact directly with the available W-tips, at low pressure (<10^−6^ torr) and at room temperature (see Methods). Fig. 2A shows a schematic of the electrical setup used to perform standard four-probe characterization. The current *I*_xx_ is forced between the outer contacts while the voltage drop *V*_ds_ is measured between the inner contacts. Fig. 2B shows the *I*–*V* characteristics measured for both four-probe (*I*_*xx*_–*V*_ds_) and two-probe (*I*_ds_–*V*_ds_) configurations resulting in a channel resistance of 885 Ω and 901 Ω, respectively. The difference between the two methods is less than 2%, indicating that the contacts are ohmic with low contact resistance.^35–37^

To perform field emission measurements, one of the tips was left in contact with the sample while the other, used as the anode, was retracted at a certain distance *d* from the surface. Fig. 2C shows a schematic of the electric setup used for the field emission measurements. The cathode–anode separation distance was precisely measured through SEM imaging by rotating the sample with respect to the electron beam to have a distance almost perpendicular to the beam. The anode W-tip has a very small radius of curvature (∼100 nm) to allow the extraction of field emission current from small areas (down to 2 μm^2^ and less) depending on the cathode–anode separation distance.^38^ Thus, the tip-shaped anode enables the probing of the field emission properties of the material with microscale spatial resolution, in contrast to other setups. The current was monitored while the anode voltage was ramped up to 100 V (a constraint imposed to avoid damage to the device and the measurement setup). Fig. 2D shows that at *d* = 200 nm and for a voltage up to ∼40 V, the current fluctuates around the noise level (∼10^−13^ A) and no clear charge flow is detected. At higher voltages, an exponential increase in the current is detected, reaching a remarkable maximum current density of ∼723 A cm^−2^, calculated considering an effective emission area of 0.12 μm^2^.^39^ This current, although lower than the one reported for individual CNTs, is several orders of magnitude higher than that reported for well-established field emitters, such as aligned CNT films (ranging from typically 0.1 up to values as large as 4 A cm^−2^)^1,40–46^ and vertically aligned graphene sheets (ranging from 10 μA cm^−2^ to 40 mA cm^−2^),^21,47–51^ demonstrating the suitability of our approach for applications in vacuum electronics. Moreover, we note that achieving vertical orientation of graphene nanosheets typically requires extremely high fabrication temperatures (up to 1000 °C)^49^ or the use of hazardous compounds,^21^ which greatly limits the feasibility of large-scale production, while the fabrication of the devices under study is carried out at room temperature and ambient pressure. Table 1 provides a comprehensive comparison between the turn-on field and maximum current densities of our device and those of vertically aligned graphene films and solution processed graphene oxide. It also includes details about the anode material and shape, anode–cathode separation distance, emission area, fabrication temperature, and chamber pressure, highlighting the advantages of our room-temperature fabrication process. Table S1† shows a comparison of the field emission characteristics of our devices with those measured for other 2D and 1D nanostructures using the same measurement setup,^52–56^ revealing that the printed graphene films under study exhibit similar turn-on fields but significantly higher maximum current densities.

**Table 1:** Comparison between the field emission figures of merit and the measurement setup used in the present work and the ones for vertical graphene nanosheets and graphene oxide

Device structure	Method	Fabrication temperature	Turn on field	Maximum current density	Anode characteristics	Anode – cathode distance	Emitting area	Pressure (mbar)	Ref.
Vertically aligned few-layer graphene	MPECVD	700 °C	1 V μm^−1^	∼10 mA cm^−2^	Stainless steel. Parallel plate setup	100 μm	—	10^−5^	47
Vertically aligned few-layer graphene	MPECVD	900 °C	1.9 V μm^−1^	17 μA cm^−2^	Cu rod (length 18 cm, diameter 1.8 cm). Parallel diode configuration	10 μm	∼8 × 10^−3^ cm^2^	2 × 10^−5^	48
Vertically oriented few-layer graphene	HFCVD	1000 °C	22 V μm^−1^	25 μA cm^−2^	Stainless steel. Hemispherical tip (radius 1 mm)	25 μm	—	∼10^−7^	49
Vertical few-layer graphene sheet	MPECVD	550 °C	1.7 V μm^−1^	7 mA cm^−2^	Planar diode method	500 μm	∼2 × 10^−1^ cm^2^	—	50
Few-layer graphene on carbon nanotubes	Radio frequency sputtering deposition	727 °C	2.5 V μm^−1^	20 mA cm^−2^	Stainless steel plate. Diode setup	1 mm	∼2 × 10^−2^ cm^2^	∼10^−7^	51
Vertical graphene sheets	Screen printing	120 °C	1.6 V μm^−1^	∼40 mA cm^−2^	Phosphor-coated ITO glass. Diode setup	500 μm	—	5 × 10^−6^	21
Reduced graphene oxide film	Screen printing	350 °C	1.5 V μm^−1^	∼3 mA cm^−2^	Phosphor-coated ITO glass. Diode setup	270 μm	—	∼10^−6^	20
Reduced graphene oxide/polystyrene composite film	Spin coating	200 °C	4 V μm^−1^	1 mA cm^−2^	—	—	—	—	19
Reduced graphene oxide film	Electrophoretic deposition	1050 °C	2.3 V μm^−1^	∼23 mA cm^−2^	Cylinder-shaped iron probe (diameter 1 mm)	100 μm	—	∼10^−7^	16
Graphene nanosheets	Inkjet printing	Room temperature	58 V μm^−1^	723 A cm^−2^	Tungsten tip (radius 100 nm)	200–800 nm	∼1 × 10^−7^ cm^2^	∼10^−6^	This work

**Fig. 3:**
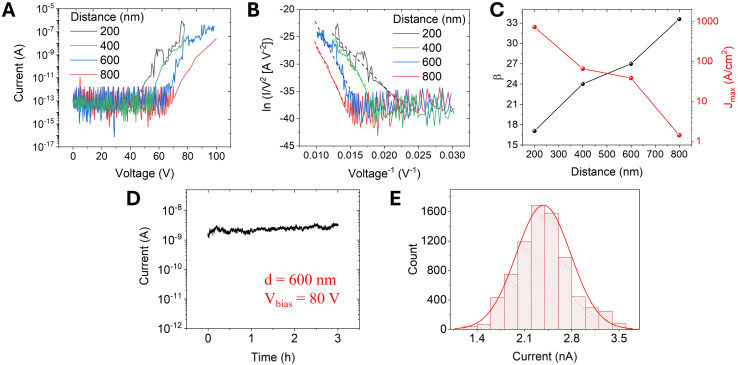
Field emission properties and analysis. (A) Field emission current as a function of the applied voltage recorded for different cathode–anode separations. (B) Fowler–Nordheim plot and superimposed linear fit used to obtain the field-enhancement factor for each distance. (C) Field enhancement factor and maximum current density as a function of the cathode–anode separation. (D) Field emission current as a function of the time measured at a cathode–anode separation distance of 600 nm and a constant bias of 80 V. (E) Histogram of measured field emission current values and Gaussian fit.

The experiment was repeated by positioning the anode tip in different positions on top of the graphene film, achieving the same field emission performance from both the centre and the edge of the film, and on three different film locations (Fig. S2†) demonstrating the possibility of extracting electrons from the whole printed films. Measurements were repeated on five independently prepared samples, all fabricated using the same ink formulation and printing protocol achieving variations in turn-on field and maximum current density within 10%. Post-field emission SEM imaging revealed no visible structural modifications to the film, confirming its strong adhesion to the substrate and mechanical robustness. To check the effect of the cathode–anode separation on the field emission properties of the devices, the W-tip was moved away from the sample surface at different distances. Fig. 3A shows the current measured for anode–cathode separation ranging from 200 nm to 800 nm. It is observed that the voltage necessary to start the field emission current, also known as turn-on field, is strongly dependent on the distance between the graphene ink and the W-tip.

In a parallel plate configuration, the electric field is simply *V*/*d*. To account for the spherical termination of the W-tip in our setup, an anode-correction factor of *k* ≈ 1.5 must be introduced^57^ and the applied electric field is expressed as *V*/*kd*. We found a turn-on field as low as 58 V μm^−1^ (defined as the field needed to obtain a current of 10 μA cm^−2^) for *d* = 800 nm, that is comparable to what has been reported for single and few layer CVD graphene and carbon nanotubes measured with the same setup.^9,11^

To confirm the field emission nature of the measured current, the *I*–*V* curves are analysed in the framework of the classical Fowler–Nordheim (FN) theory.^58^ Although this model was originally derived for the electronic emission from a flat metallic surface through a triangular potential barrier at zero kelvin, it is a common model to get a first-approximation understanding of the emission properties from several nanostructures. According to the Fowler–Nordheim model, the field emission current can be expressed as1
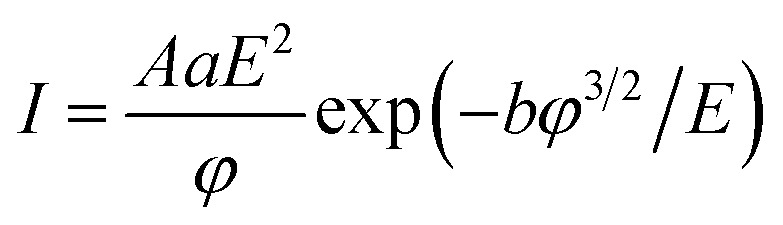
where *A* is the emitting area, *E* is the local electric field, *φ* is the work function of the material (4.3 eV for graphene^59^), and *a* = 1.54 × 10^−6^ A eV V^−2^ and *b* = 6.83 × 10^9^ V m^−1^ eV^−3/2^ are constants. We note that, due to the disordered nature of the printed films and the residual chemicals adsorbed, parameters such as work function may differ significantly from those of single-crystal graphene. The value reported here is based on experimental measurements from our previous study on graphene–silicon Schottky diodes fabricated using the same printed graphene films. A local amplification factor needs to be introduced since the applied field, owing to the accumulation of the field lines at the apex of the emitting site, can be enhanced by several orders of magnitude. This amplification factor is usually indicated as *β* and allows the local field *E* to be expressed as: 2
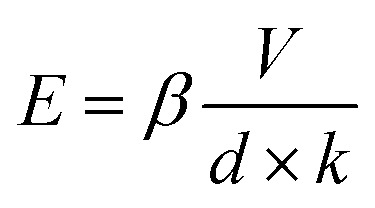


The field enhancement factor *β* constitutes an important figure of merit in field emission studies, being strictly dependent on the shape of the emitter. From eqn (2), it is possible to obtain *β* from a simple linear fit of the ln(*I*/*V*^2^) *versus* 1/*V* plot (Fowler–Nordheim plot). Fig. 3B shows the obtained curves with the superimposed linear fits that have been used to obtain the field-enhancement factor through the slope [*m* = (*bφ*^3/2^*dk*)/*β*]. Fig. 3C displays *β* and the maximum current density for different cathode–anode distances. The effective area of emission has been calculated through finite element electrostatic simulations of the electric field generated by the applied potential difference between the tip and the sample, following the procedure reported in a previous study.^60^ We found that *β* increases with the cathode–anode distance, consistently with what has been reported for field emission from carbon nanotubes^9^ or nanowires,^61^ while the max current density decreases with cathode–anode separation. The observed decrease in current density is attributed to a reduction in local electric field intensity due to the wider spread of field lines over a larger effective emission area, as supported by finite-element simulations and consistent with prior studies.^62,63^ Our measurements found that *β* ∼35 for *d* = 800 nm and the turn on field is 58 *V* μm^−1^. Of note is that the high turn-on field and the relatively small *β*, as compared to values reported for r-GO,^16,19,20^ may not be related to differences in materials properties, but they could be attributed to the specific measurement setup used in this work, which is based on an anode tip with a very small radius of curvature positioned at a short distance from the cathode to specifically probe field emission from localized regions of the films. This is in contrast to previous studies that employed larger tips positioned at longer distances (up to 1 mm radius of curvature and 100 μm anode–cathode separation),^16^ facilitating electron extraction from larger areas of the film at lower bias. We report a remarkable max current density of ∼723 A cm^−2^ that makes our inkjet printed graphene films very attractive for large area field emission devices. When comparing the field-emission parameters of our printed graphene films to those obtained in previous studies using our identical setup (Table S1†), we observe that, as expected, our films exhibit a slightly lower turn-on field compared to planar single flakes, but a slightly higher turn-on field compared to 1D nanostructures. Furthermore, the maximum current density, evaluated over a similar emission area, is significantly higher, confirming the superior field emission performance of our printed graphene films. Finally, the FE current stability is investigated. A tungsten tip was positioned in contact with the graphene film to serve as the cathode, while another tungsten tip, acting as the anode, was retracted to a fixed distance of 600 nm. A constant bias of 80 V was applied to the anode, and the field emission current was monitored over time, following an initial electrical conditioning to desorb adsorbates. Fig. 3D shows the field emission current over time revealing stable emission for at least 3 hours. Statistical analysis of the measured current values, presented as a histogram in Fig. 3E, exhibits a Gaussian distribution centered around an average value of 2.4 nA with a standard deviation of 0.4 nA, corresponding to a variability <17%. Such stability time is comparable to the ones measured from other solution processed graphene films.^16,20,21^

## Conclusions

We demonstrated the suitability of water-based graphene ink made by LPE and deposited *via* inkjet printing to fabricate field emitters on a large scale. Our fabrication approach is conducted entirely at room temperature, eliminating the need for vacuum conditions and enabling the efficient, cost-effective production of large-area emitters. We reported field emission performance with a relatively high field-enhancement factor and a remarkable maximum current density of ∼723 A cm^−2^ stable for at least 3 hours. Our results demonstrate that the graphene inkjet printed films are suitable for a wide range of applications in vacuum electronics, paving the way to a new era of large area field emitting devices.

## Materials and methods

### Materials

Bulk graphite powder was purchased from Graphexel or Sigma-Aldrich (99.5% grade). Doped n-Si (100) wafers with a resistivity of ∼10 Ω cm, corresponding to a phosphorus dopant density of ∼4.5 × 10^14^ cm^−3^, covered with 300 nm thermally grown SiO_2_, were purchased from Siegert Wafer. Silver conductive paste was sourced from Sigma-Aldrich.

### Ink preparation and printing

The water-based inks were prepared as described in ref. 30. A Varian Cary 5000 UV-Vis spectrometer has been used to measure the absorption spectrum of the ink. The Lambert–Beer law has been used to obtain the concentration of graphene, by using an absorption coefficient of 2460 measured at 660 nm.^64^ Immediately before printing the Si substrates have been cleaned with acetone, isopropanol and DI water and then treated with Ar plasma for 15 s to improve printability. A Dimatix Materials Printer 2850 (Fujifilm) was used to print 2 mg mL^−1^ graphene ink with a length *L* of 5 mm and width *W* of 0.5 mm (see Fig. 1a). The spatial resolution of the printer is of ∼50 μm on silicon. The platen temperature was set at 45 °C and cartridges with a droplet volume of 10 pL were used setting a drop spacing of 35 μm and 80 printing passes.

### Film characterization

For the Raman measurements, a Renishaw Invia Raman spectrometer, equipped with a laser with an excitation wavelength of 514.5 nm and 2 mW laser power, a 100× NA, 0.85 objective lens and a 2400 grooves per mm grating, was used. For the streamline mapping, an area of 1 × 0.2 mm^2^ has been selected and Raman measurements have been carried out with 10 μm steps. The graphene coverage was determined by generating Raman maps of the G and 2D peak intensity. A Veeco Dektak 8 Stylus profilometer characterized by a stylus force of 3 mg and a scan resolution of ∼0.28 μm was used to measure the roughness of the substrate and the thickness of the printed graphene.

### Electrical measurements

Electrical measurements were performed with a Janis Probe Station (Janis ST-500 probe station) equipped with four nanoprobes connected to a Keithley 4200 SCS (semiconductor characterization system), working as a source-measurement unit with current sensitivity better than 1 pA. Field emission measurements were performed inside a ZEISS, LEO 1430 SEM chamber, at low pressure, <10^−6^ torr, and at room temperature. Two W-tips (MM3A-nanoprobe system by Kleindeik Company), with positioning controlled with a resolution better than 5 nm in all directions, were used as the anode and the cathode, respectively. The two tips were electrically connected by means of triaxial feedthroughs to an external semiconductor parameter analyser (Keithley 4200-SCS).

## Author contributions

A. G. conceived the study and designed the experiments, under the supervision of C. C.; T. A. prepared the graphene ink and performed the profilometry measurements, while fabrication of the devices was conducted by A. G.; J. W. performed the Raman measurements; A. P. and D. C. performed the electrical characterization, under the supervision of A. D. B. and M. P.; E. F. performed the stability measurements under the supervision of M. P.; A. G. wrote the manuscript with contributions from all authors. All the authors have discussed the results and provided comments regarding the manuscript.

## Conflicts of interest

The authors declare no competing financial interest.

## Supplementary Material

NA-OLF-D5NA00161G-s001

NA-OLF-D5NA00161G-s002

NA-OLF-D5NA00161G-s003

NA-OLF-D5NA00161G-s004

NA-OLF-D5NA00161G-s005

## Data Availability

The data supporting this article are included within the manuscript and as part of the ESI.†
