# Supplementary figures and images for: Electron field emission of water-based inkjet printed graphene films
Source: Nanoscale Adv. 2025 Jun 24;7(17):5184–92. doi: 10.1039/d5na00161g (PMC12262138; doi:10.1039/d5na00161g)

**A**

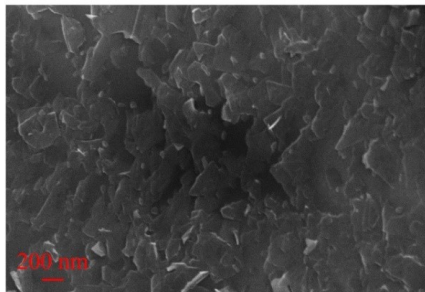

**B**

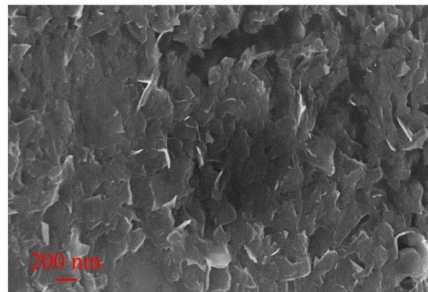

**C**

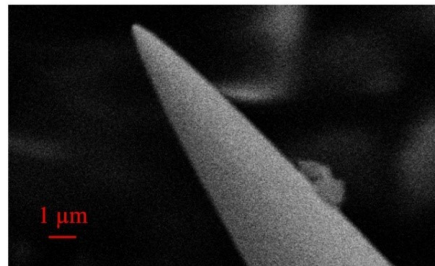

Supplement: NA-007-D5NA00161G-s002 [file NA-007-D5NA00161G-s002.pdf]

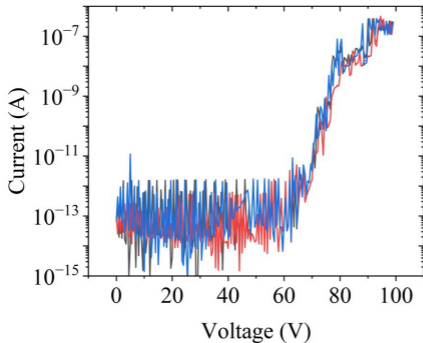

Supplement: NA-007-D5NA00161G-s003 [file NA-007-D5NA00161G-s003.pdf]
