# Supplementary material for: Electron field emission of water-based inkjet printed graphene films
Source: Nanoscale Adv. 2025 Jun 24;7(17):5184–92. doi: 10.1039/d5na00161g (PMC12262138; doi:10.1039/d5na00161g)
Supplement: NA-007-D5NA00161G-s004 [file NA-007-D5NA00161G-s004.pdf]

Supplementary Information (SI) for Nanoscale Advances.
This journal is © The Royal Society of Chemistry 2025

Supporting Information

Electron Field Emission of Water-based Inkjet Printed Graphene Films

Alessandro Grillo[1,], Towseef Ahmad[1], Jingjing Wang[1], Aniello Pelella[2], Enver Faella[4], Daniele Capista[3], Maurizio Passacantando[4], Antonio Di Bartolomeo[2], Cinzia Casiraghi[1,*]*

[1] *Department of Chemistry, University of Manchester, Manchester, M13 9PL, UK*

[2] *Physics Department "E. R. Caianiello", University of Salerno, via Giovanni Paolo II n. 132, Fisciano 84084, Salerno, Italy*

[3] *IHP, Im Technologiepark 25, 15236 Frankfurt (Oder), Germany*

[4] *Dipartimento di Scienze Fisiche e Chimiche, Università degli Studi dell'Aquila, Via Vetoio, 67100 Coppito (AQ), Italy*

*Corresponding authors email: cinzia.casiraghi@manchester.ac.uk; alessandro.grillo@manchester.ac.uk

I Literature comparison

II Additional SEM images

III Additional electrical measurements

Device Structure	*Method*	*Fabrication Temperature*	*Turn on field*	*Maximum Current Density*	*Anode characteristics*	*Anode – cathode distance*	*Emitting area*	*Pressure (mbar)*	*Ref.*
Graphene on carbon fiber	CVD	/	70 V/µm	12 A/cm^2	Tungsten tip (radius 100 nm)	400-700 nm	$8 \cdot 10^{-8}$ cm^2	$\sim 10^{-6}$	1
MoS_2 monolayer	CVD	750 °C	100 V/µm	0.2 A/cm^2	Tungsten tip (radius 100 nm)	200-400 nm	$\sim 1 \cdot 10^{-7}$ cm^2	$\sim 10^{-6}$	2
Few layers GeAs	Mechanical Exfoliation	/	80 V/µm	10 A/cm^2	Tungsten tip (radius 100 nm)	400 nm	$\sim 1 \cdot 10^{-7}$ cm^2	$\sim 10^{-6}$	3
Few layers $PdSe_2$	Mechanical Exfoliation	850 °C	60 V/µm	10 A/cm^2	Tungsten tip (radius 100 nm)	70-300 nm	$\sim 1 \cdot 10^{-7}$ cm^2	$\sim 10^{-6}$	4
β-Ga_2O_3 nanopillars	Ion Etching	/	30 V/µm	100 A/cm^2	Tungsten tip (radius 100 nm)	400-1000 nm	$\sim 4 \cdot 10^{-8}$ cm^2	$\sim 10^{-6}$	5
Graphene nanosheets	Inkjet Printing	Room Temperature	58 V/µm	723 A/cm^2	Tungsten tip (radius 100 nm)	200-800 nm	$\sim 1 \cdot 10^{-7}$ cm^2	$\sim 10^{-6}$	This work

I Literature comparison

Table S1. Comparison of the field emission figure of merits of the graphene film measured in this work with those of 2D and 1D nanostructures measured in the same experimental

conditions.

II Additional SEM images

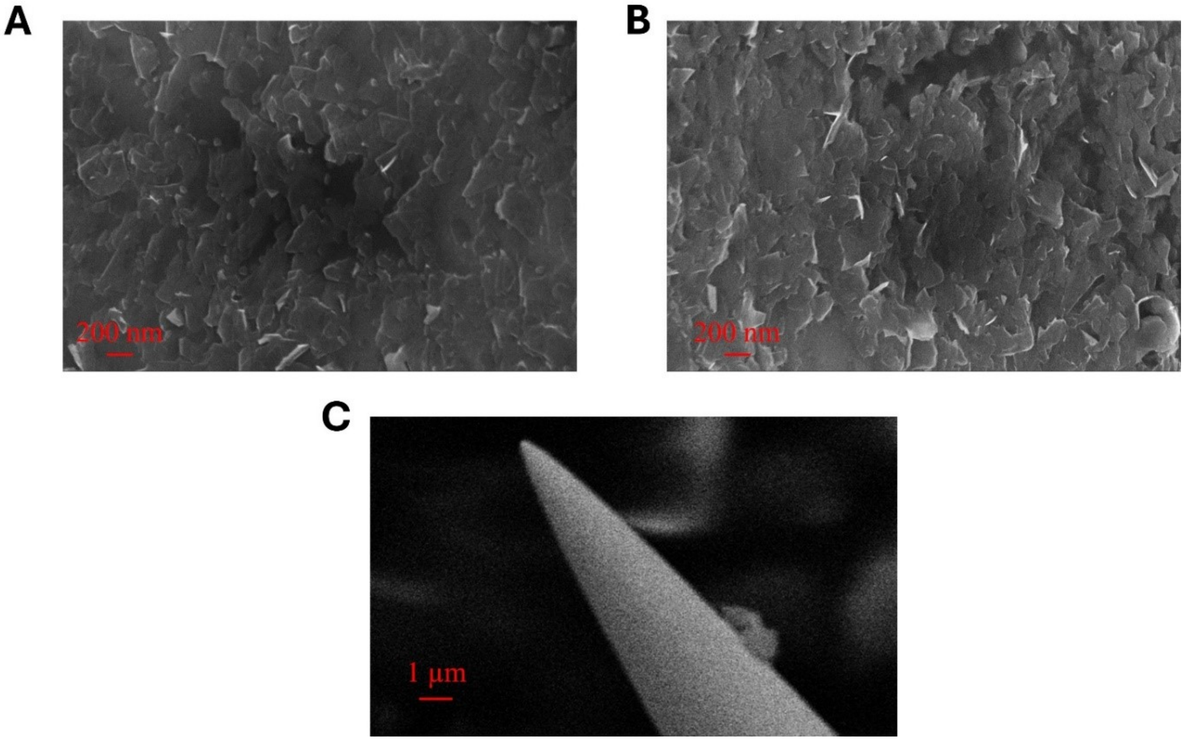

Figure S1 – 45° (A) and 60° (B) tilted SEM images of the printed graphene film. C) SEM image of the W-tip used for the field emission measurements.

III Additional electrical measurements

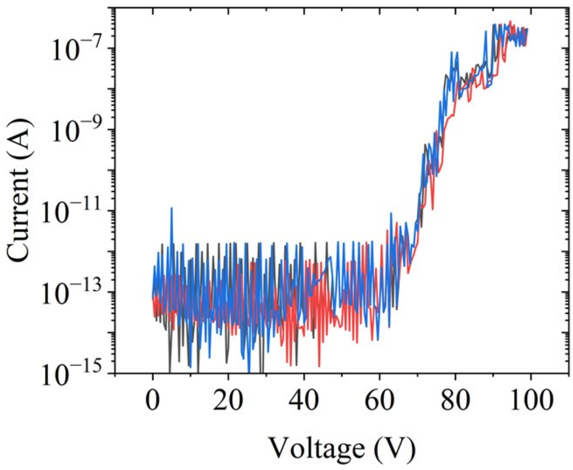

Figure S2 – I-V curve recorded with the field emission setup at three different points on the film and at a fixed anode-cathode separation of 400 nm.

References

1. Giubileo, F. *et al.* Local Characterization of Field Emission Properties of Graphene Flowers. *Adv Elect Materials* **9**, 2200690 (2023).

2. Pelella, A. *et al.* Gate-Controlled Field Emission Current from MoS_2 Nanosheets. *Adv Elect Materials* **7**, 2000838 (2021).

3. Di Bartolomeo, A. *et al.* Field emission from two-dimensional GeAs. *J. Phys. D: Appl. Phys.* **54**, 105302 (2021).

4. Di Bartolomeo, A. *et al.* Field Emission in Ultrathin $PdSe_2$ Back-Gated Transistors. *Adv Elect Materials* **6**, 2000094 (2020)

5. Grillo, A. *et al.* High field-emission current density from β-Ga2O3 nanopillars. *Applied Physics Letters* **114**, 193101 (2019).
