# Supplementary material for: Electron field emission of water-based inkjet printed graphene films
Source: Nanoscale Adv. 2025 Jun 24;7(17):5184–92. doi: 10.1039/d5na00161g (PMC12262138; doi:10.1039/d5na00161g)
Supplement: NA-007-D5NA00161G-s005 [file NA-007-D5NA00161G-s005.pdf]

Supplementary Information (SI) for Nanoscale Advances.
This journal is © The Royal Society of Chemistry 2025

Device Structure	*Method*	*Fabrication Temperature*	*Turn on field*	*Maximum Current Density*	*Anode characteristics*	*Anode – cathode distance*	*Emitting area*	*Pressure (mbar)*	*Ref.*
Graphene on carbon fiber	CVD	/	70 V/µm	~12 A/cm^2	Tungsten tip (radius 100 nm)	400-700 nm	$8\cdot10^{-8}$ cm^2	~ 10^{-6}	1
MoS_2 monolayer	CVD	750 °C	100 V/µm	0.2 A/cm^2	Tungsten tip (radius 100 nm)	200-400 nm	~ $1\cdot10^{-7}$ cm^2	~ 10^{-6}	2
Few layers GeAs	Mechanical Exfoliation	/	80 V/µm	10 A/cm^2	Tungsten tip (radius 100 nm)	400 nm	~ $1\cdot10^{-7}$ cm^2	~ 10^{-6}	3
Few layers $PdSe_2$	Mechanical Exfoliation	850 °C	60 V/µm	10 A/cm^2	Tungsten tip (radius 100 nm)	70-300 nm	~ $1\cdot10^{-7}$ cm^2	~ 10^{-6}	4
β-Ga_2O_3 nanopillars	Ion Etching	/	30 V/µm	100 A/cm^2	Tungsten tip (radius 100 nm)	400-1000 nm	~ $4\cdot10^{-8}$ cm^2	~ 10^{-6}	5
Graphene nanosheets	Inkjet Printing	Room Temperature	58 V/µm	723 A/cm^2	Tungsten tip (radius 100 nm)	200-800 nm	~ $1\cdot10^{-7}$ cm^2	~ 10^{-6}	This work
